# Diet-Driven Microglial Activation: Region-Specific Neuroinflammation in the Mouse Brain

**DOI:** 10.3390/brainsci16010029

**Published:** 2025-12-25

**Authors:** Laura Plantera, Stephan H. Bernhart, Kerstin Immig, Judith Leyh, Uta Ceglarek, Ingo Bechmann

**Affiliations:** 1 Institute of Anatomy, Faculty of Medicine, Leipzig University, 04103 Leipzig, Germanyingo.bechmann@medizin.uni-leipzig.de (I.B.); 2 Interdisciplinary Centre for Bioinformatics, Leipzig University, 04107 Leipzig, Germany; 3 Institute of Laboratory Medicine, Clinical Chemistry and Molecular Diagnostics, Faculty of Medicine, Leipzig University, 04103 Leipzig, Germany

**Keywords:** obesity, microglia, neuroinflammation, NF-κB, Alzheimer’s disease

## Abstract

**Background**: High-fat diet (HFD) consumption is commonly linked to low-grade brain inflammation and increased risk of neurodegeneration. However, in our previous research, HFD exposure for up to 24 weeks did not increase pro-inflammatory cytokine expression or impair learning and spatial memory. To further investigate neuroimmune responses, we examined microglial activation at the transcriptional level. **Methods**: Male C57BL/6J mice were fed either a normal diet (ND) or HFD for 4, 12, or 24 weeks. Bulk RNA sequencing was performed across four brain regions (cerebellum, hippocampus, hypothalamus, cortex) to assess region-specific transcriptional responses. **Results**: HFD induced region- and time-dependent transcriptional changes. In the hypothalamus, 0/11/37 differentially expressed genes (DEGs; *p*-value < 0.05; fold change > 1.5) were detected at 4, 12, and 24 weeks, respectively. In the hippocampus, 2/41/42 DEGS were observed; in the cortex, 1/3/68 DEGS; and in the cerebellum, 27/0/0 DEGS at the respective time points, indicating minimal cerebellar involvement beyond the early time point. Across all conditions, three genes (*Lcn2*, *Ch25h*, *Gimap9*) were consistently regulated. Several DEGs were linked to microglial activation and inflammatory signaling. In the manuscript, we discuss 33 biologically relevant DEGs in detail. Transcriptomic signatures and pathway enrichment analyses suggest potential engagement of NF-κB-related pathways, although this interpretation remains indirect. **Conclusions**: These findings demonstrate that HFD selectively alters brain homeostasis by inducing region-specific transcriptional changes associated with microglial activation and inflammatory processes. While NF-κB-related pathways emerged as recurrent candidates, direct mechanistic validation is required.

## 1. Introduction

Obesity is characterized by chronic, low-grade systemic inflammation [1,2,3,4,5,6]. Diet-induced obesity, in particular, has been linked to cognitive impairment and elevated risk of neurodegenerative diseases, including Alzheimer’s disease (AD), due to its capacity to promote brain inflammation and accelerate neuroaging [7]. Microglia, the brain’s resident immune cells, constitute the first line of defense against injury and pathology [8]. These highly specialized cells continuously monitor the microenvironment for tissue damage, infection, or homeostatic imbalance [9].

Microglia’s efficacy is affected by aging [10]. Age-related senescence impairs their neuroprotective function, leaving neurons vulnerable to degeneration because they lack the microglial support necessary for their survival [11]. These changes develop gradually over decades and are likely the consequence of various influences on the central nervous system (CNS) microenvironment, particularly oxidative damage caused by free radicals [12]. However, microglial pathology can also occur as a result of sudden extreme stress, as findings from experimental animal studies show [12,13,14].

Regardless of whether triggered by aging or acute stress, microglial activation is accompanied by structural remodeling and secretion of inflammatory mediators. These include cytokines (TNF-α, IL-1β, IL-6), chemokines (CCL2), reactive oxygen species (ROS), nitric oxide (NO), and matrix metallopeptidase 12 (MMP-12), all of which contribute to inflammation, neuronal loss, and tissue damage [15]. In seeking to research their activation state, studies on humans and rodent models have found that microglia respond to a high-fat diet (HFD), leading to acute microgliosis in hypothalamic regions responsible for the regulation of nutrition and metabolism [16,17]. Microglial activation in the hypothalamus of mice after a long-term HFD has also been observed in previous studies conducted by our research group [18].

A central mediator linking neuroinflammation and lipid metabolism is nuclear factor kappa B (NF-κB), a transcription factor that regulates numerous inflammatory processes [19]. It is clear that NF-κB signaling does not occur in isolation in the regulation of numerous physiological and pathological processes in which it is involved. However, it is less clear whether there might be direct or indirect regulation with other molecules. Consequently, this regulation would trigger interactions with various signaling pathways, including the NF-κB-STAT3 loop, PI3K/AKT pathway, and the TLR4/NF-κB pathway [20,21]. These crosstalk mechanisms amplify inflammatory responses.

Once activated, the first of these signaling pathways, the NF-κB and the signal transducer and activator of transcription 3 (STAT3)-loop, regulates the expression of anti-apoptotic, pro-proliferative, and immune response genes. Some of these genes overlap and require transcriptional cooperation between the two factors [22]. This positive feedback loop acts as an epigenetic switch, transforming transient inflammatory signals into a chronic inflammatory state maintained by NF-κB activity [23].

The second pathway is essential for maintaining energy balance through the integrity of the phosphatidylinositol 3′–kinase (PI3K)/serine/threonine protein kinase protein kinase B (AKT) pathway. Thus, it plays a crucial role in the development of obesity and obesity-related metabolic disorders, either on the level of tissue inflammation or in the regulation of energy homeostasis. The PI3K protein family is responsible for abnormal metabolic disease downstream of PI3K in the glucose, insulin, and lipid signaling pathways [24]. Depending on context, modulation of PI3K/AKT can either alleviate or worsen obesity-associated outcomes.

Finally, the toll-like receptor 4 (TLR4) signaling pathway is considered one of the main triggers of the obesity-induced inflammatory response [25]. There is evidence that saturated fatty acids can also bind to TLR4 and activate TLR4-mediated signaling [26], leading to enhanced NF-κB activity.

In summary, activation of the described signaling pathways increases the expression of pro-inflammatory cytokines, such as tumor necrosis factor alpha (TNF-α), interleukin-1 (IL-1), and interleukin-6 (IL-6), by stimulating the transcription factor NF-κB. It is worth noting that NF-κB itself regulates the activation, differentiation, and effector function of inflammatory T cells [27] and regulates the initiation of inflammasomes [28]. Deregulated NF-κB activation leads to chronic inflammatory diseases.

Obesity is associated with neuroinflammation through diet-induced microglial activation. In this study, we investigated the effects of HFD on indicators of microglial activation across multiple brain regions (cerebellum, hippocampus, hypothalamus, cortex) in male C57BL/6J mice. Using bulk RNA sequencing at 4, 12, and 24 weeks, we observed time-dependent differentially expressed genes (DEGs), including several associated with microglial activation and inflammatory pathways potentially involving NF-κB-mediated signaling. However, direct mechanistic verification will be required in future studies.

## 2. Materials and Methods

### 2.1. Animals and Diets

Male C57BL/6J wild-type mice were obtained from Janvier Labs (Le Genest-Saint-Isle, France) at the age of six weeks. They had two weeks of acclimatization before the start of the experiment. They were housed in the local animal facility under standard conditions: 12 h dark/light cycle, group-housed with free access to water and food. Mice (8 weeks old) were assigned to either a normal diet (ND) (11 kcal% fat, 53 kcal% carbohydrates, 36 kcal% protein; V1124-300, ssniff Spezialdiäten GmbH, Soest, Germany) or an HFD (59 kcal% fat, 26 kcal% carbohydrates, 15 kcal% protein; E15772-34, ssniff Spezialdiäten GmbH, Soest, Germany) for 4, 12, or 24 weeks. The diet composition of the HFD is provided in the Supplementary Material (Supplementary Data S4). Body weight was measured weekly during the whole experiment. All experimental procedures and protocols were authorized by the local ethics committee of the state of Saxony (Landesdirektion Sachsen, Leipzig, approval no. TVV 41/17, approval date 7 February 2018). We performed this study in accordance with the guidelines of the Animal Experimental Committee following the German Animal Welfare Act as well as the European guidelines (Directive 2010/63/EU) concerning the protection of laboratory animals.

### 2.2. Tissue Preparation

At the end of each feeding period, mice were anesthetized with isoflurane (Baxter GmbH, Unterschleißheim, Germany) and subsequently perfused with ice-cold PBS (Gibco, Life Technologies, Darmstadt, Germany) to clear the intravascular compartment of the brain from blood cells. The hypothalamus, hippocampus, cerebellum, and frontal cortex were quickly dissected on ice (72 samples total: 6 groups × 3 animals per group × 4 brain regions) (Table 1). Brain tissue was flash-frozen in liquid nitrogen and stored at −80 °C until RNA extraction.

### 2.3. RNA Extraction

Total RNA was isolated from the hypothalamus, hippocampus, cerebellum, and prefrontal murine cortices using the RNeasy Mini Kit (Qiagen, Hilden, Germany) according to the manufacturer’s instructions. Samples were stabilized in RNAprotect buffer (Qiagen, Hilden, Germany) and stored at 2–8 °C until processing.

### 2.4. RNA Sequencing

RNA library preparation and RNA sequencing were performed at the Genomics Core Facility “KFB—Center of Excellence for Fluorescent Bioanalytics” (University of Regensburg, Regensburg, Germany; www.kfb-regensburg.de; accessed on 5 November 2021). Library preparation and RNAseq were carried out as described in the Illumina “Stranded mRNA Prep Ligation” Reference Guide, the Illumina NextSeq 2000 Sequencing System Guide (Illumina, Inc., San Diego, CA, USA), and the KAPA Library Quantification Kit—Illumina/ABI Prism (Roche Sequencing Solutions, Inc., Pleasanton, CA, USA).

In brief, 200 ng of total RNA was used for purifying the poly-A-containing mRNA molecules using oligo(dT) magnetic beads. Following purification, the mRNA was fragmented to an average insert size of 200–400 bases using divalent cations under elevated temperature (94 °C for 8 min). Next, the cleaved RNA fragments were reverse-transcribed into first-strand complementary DNA (cDNA) using reverse transcriptase and random hexamer primers. Thereby, Actinomycin D was added to allow RNA-dependent synthesis and to improve strand specificity by preventing spurious DNA-dependent synthesis. Blunt-ended second-strand cDNA was synthesized using DNA Polymerase I, RNase H, and dUTP nucleotides. The incorporation of dUTP, in place of dTTP, quenches the second strand during the later PCR amplification because the polymerase does not incorporate past this nucleotide. The resulting cDNA fragments were adenylated at the 3′ ends, and the pre-index anchors were ligated. Finally, DNA libraries were created using a 13-cycle PCR to selectively amplify the anchor-ligated DNA fragments and to add the unique dual indexing (i7 and I5) adapters. The libraries were bead-purified twice and quantified using the KAPA Library Quantification Kit. Equimolar amounts of each library were sequenced on an Illumina NextSeq 2000 instrument controlled by the NextSeq 2000 Control Software (NCS) v1.4.1.39716, using two 50-cycle P3 Flow Cells with the dual index, single-read (SR) run parameters. Image analysis and base calling were done by the Real Time Analysis Software (RTA) v3.9.25. The resulting .cbcl files were converted into .fastq files with the bcl2fastq v2.20 software.

### 2.5. Differential Gene Expression Analysis and Data Visualization

Raw reads were processed with Monsda v1.2.0 [29] using the following workflow: adapter and quality trimming with trim_galore v0.6.7 [30] and cutadapt v4.5 [31] with a quality cut-off of 15, a minimum length of 8, and an adapter error rate of 0.15, using automated recognition of adapter sequences. Quality control was assessed with FastQC and MultiQC. Reads were aligned to the mouse genome GRCmm39 (mm10; https://www.ncbi.nlm.nih.gov/datasets/genome/GCF_000001635.27/; accessed on 17 November 2023) using STAR v2.7.10b [32] and the Gencode vM33 annotation (https://ftp.ebi.ac.uk/pub/databases/gencode/Gencode_mouse/release_M33/; accessed on 17 November 2023) with the sjdbGTFfeatureExon “exon” and --sjdbGTFtagExonParentTranscript “Parent” options. Gene counts were obtained with featureCounts [33], a part of the subread package (https://subread.sourceforge.net/; accessed on 17 November 2023), strand-specific, against gencode vM33, counting all overlapping meta features (-O) and fractionally counting multi-mapping reads (-M --fraction flags). The mapped data on which this manuscript is based have been deposited in the European Nucleotide Archive (ENA) (https://www.ebi.ac.uk/ena/browser/home; accessed on 11 November 2025) under the project accession PRJEB104708.

Differential expression (DE) analysis was conducted in R v4.1.3 using DESeq2 v1.34 [34]. Technical replicates were treated as a single dataset in DE analysis by using the mean expression of the two replicates. STRING-database [35] analysis was done using the Application Programming Interface (API) with an in-house Python v3.12 script. Networks were generated on the string-db.org homepage and recolored by hand. Volcano plots were generated using the EnhancedVolcano v1.26.0 [36] R package. We used adjusted *p*-values below 0.05 and an absolute log2 fold change above 0.585 (corresponding to a fold change of 1.5) as thresholds to define DEGs. Principal Component Analyses (PCAs) were generated from DESeq2-normalized expression values using the ggplot2 v4.0.1, ggfortify v0.4.19 [37], and plotly v4.11.0 [38] R-packages.

Heatmap and expression correlations were computed in R based on DESeq2-normalized read counts. Correlations were plotted using the corrplot v0.95 [39] R package. Heatmaps were constructed using heatmap.2 from the gplots v3.3.0 [40] R package. One heatmap was constructed of all 216 genes that had an adjusted *p*-value below 0.05 and an absolute log2fold change above 0.585 in any of the regional DE analysis at any time (hypothalamus, hippocampus, cerebellum, or cortex at 4, 12, or 24 weeks) or the full HFD vs. ND analysis (Supplementary Figure S1). Another heatmap was constructed from all genes that were mentioned in the text of the manuscript (Supplementary Figure S2).

## 3. Results

### 3.1. Body Weight and Weight Gain

**Figure 1:**
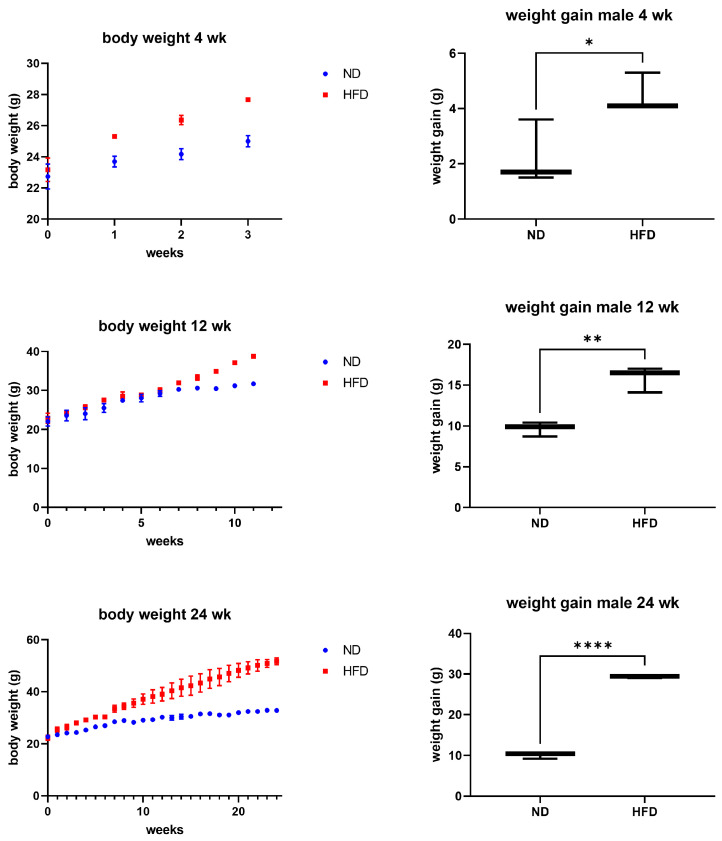
Body weight and weight gain. After different periods of feeding (4, 12, 24 weeks), ND- or HFD-fed mice (*n* = 3 per group) showed significantly increased weight gain in the HFD groups after all time periods examined. Unpaired *T*-test, *p*-value < 0.05 (* *p* < 0.05, ** *p* < 0.01, and **** *p* < 0.0001).

Male mice fed an HFD displayed a clear and consistent increase in both body weight and weight gain compared to ND-fed controls across all feeding periods (4, 12, and 24 weeks) (Figure 1). The effect was already apparent after four weeks and persisted throughout the experiment, confirming that the diet reliably induced an obese phenotype.

### 3.2. Global Transcriptional Variation

**Figure 2:**
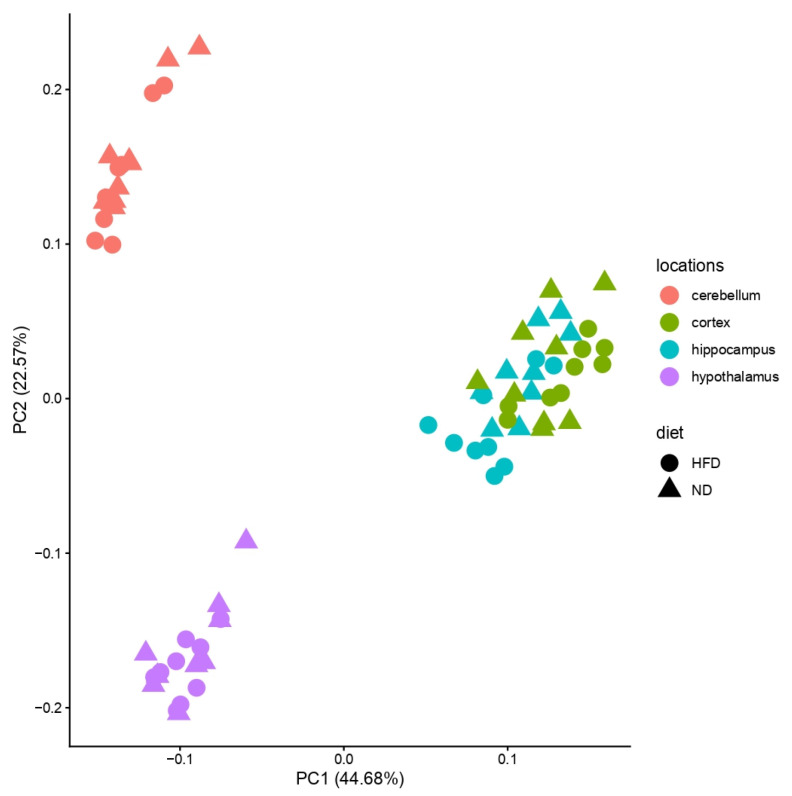
Principal Component Analysis (PCA) of all brain regions. *X*-axis: variance in the data; *y*-axis: second most variance; each point represents a sample. PCA of the four analyzed brain regions (hypothalamus—purple, hippocampus—blue, cerebellum—red, cortex—green) showed separation for cerebellum and hypothalamus, but cortex and hippocampus clustered together. Samples of HFD-fed mice are shown with circles and samples of ND-fed mice with triangles.

**Figure 3:**
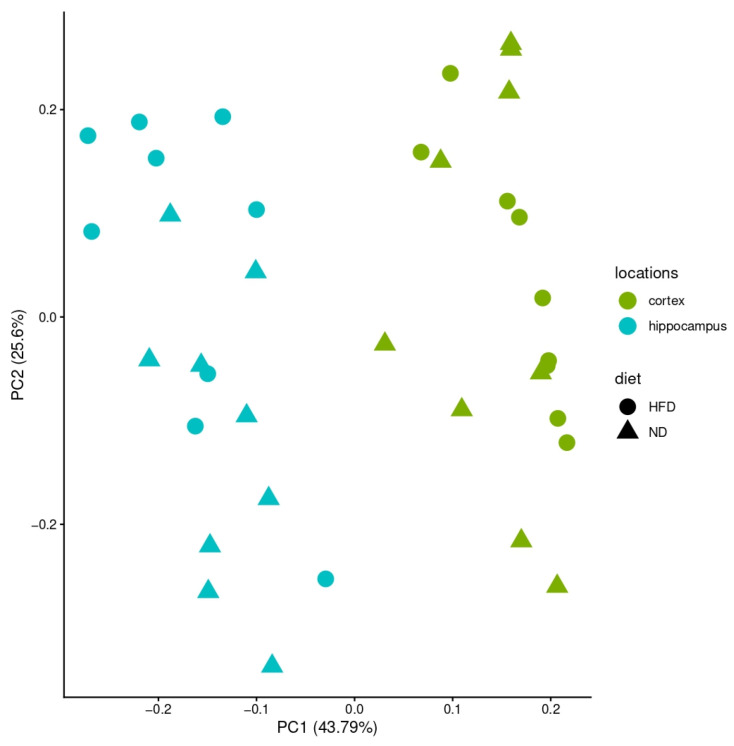
PCA of hippocampus and cortex. *X*-axis: variance in the data; *y*-axis: second most variance; each point represents a sample. The two analyzed brain regions—hippocampus (blue) and cortex (green)—can be separated when analyzed alone. Samples of HFD-fed mice are shown with circles and samples of ND-fed mice with triangles.

Bulk RNA sequencing was conducted in four brain regions (cortex, cerebellum, hypothalamus, hippocampus) across three feeding periods (4, 12, 24 weeks) and two diets (ND, HFD), yielding 72 samples in total. Principal component analysis (PCA) of all samples revealed strong separation by brain region, with the cerebellum and hypothalamus forming distinct clusters, while cortex and hippocampus overlapped (Figure 2 and Figure 3).

**Figure 4:**
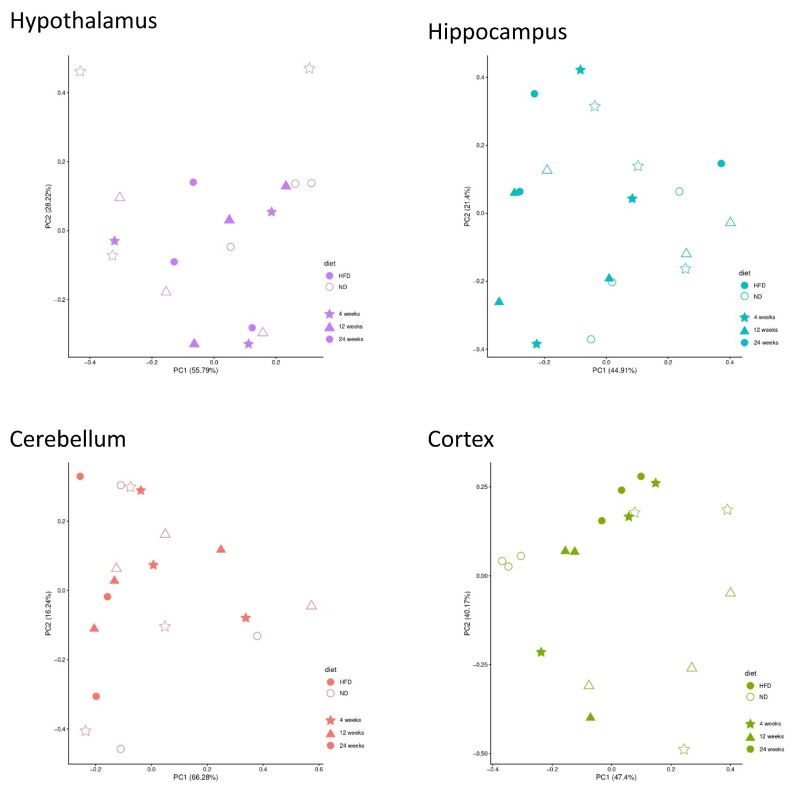
PCA separated by the specific brain regions. *X*-axis: variance in the data; *y*-axis: second most variance; each point represents a sample. Hypothalamus—purple, hippocampus—blue, cerebellum—red, and cortex—green. Samples of HFD-fed mice are displayed as filled symbols and samples of ND-fed mice as open symbols. Shapes indicate the feeding duration: star = 4 weeks, triangle = 12 weeks, circle = 24 weeks. PCA within each brain region indicated that variance was not primarily driven by diet or feeding duration.

When analyzed separately by brain region, most of the observed variance was unrelated to diet or feeding duration (Figure 4). All DEGs are listed in the Supplementary Data S1–S3.

### 3.3. Differentially Expressed Genes

**Figure 5:**
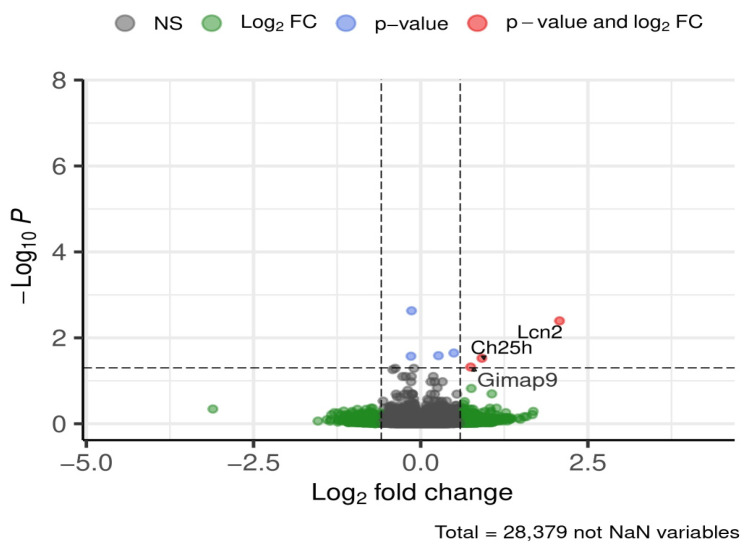
DEGs global (all brain regions, all time periods). Dashed lines mark thresholds for significance (horizontal) and fold-change (vertical).

Across all brain regions and time periods, three genes were consistently differentially expressed between ND and HFD groups (Figure 5).

Lipocalin-2 (*Lcn2*) expression was approximately fourfold higher in ND compared to HFD. Cholesterol 25-hydroxylase (*Ch25h*) expression was nearly doubled in ND relative to HFD, independent of brain region or feeding period. GTPase, IMAP family member 9 (*Gimap9*) was also upregulated under ND conditions.

When brain regions and time points were examined separately, additional significantly regulated genes (adjusted *p*-value < 0.05, fold change > 1.5) were identified and are presented in detail in the following sections. Among these significant DEGs, we selected a subset of genes of interest for detailed discussion based on their known involvement in microglial activation and inflammatory pathways. Supplementary Figures S1–S3 provide an overview of these findings: a comparative heatmap of all genes and samples (Supplementary Figure S1), a heatmap of the 33 genes of interest discussed below (Supplementary Figure S2), and a correlation matrix of all samples (Supplementary Figure S3), highlighting both shared and region-specific transcriptional patterns.

#### 3.3.1. Hypothalamus

**Figure 6:**
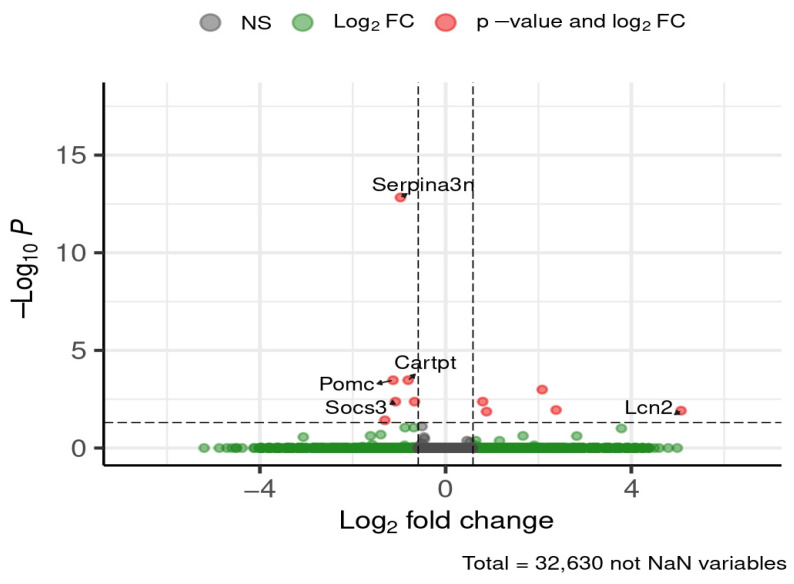
Selected DEGs of interest in the hypothalamus after 12 weeks of diet. Dashed lines mark thresholds for significance (horizontal) and fold-change (vertical).

Following 12 weeks of diet exposure, five genes central to neuroendocrine regulation (*Pomc*, *Cartpt*) and inflammatory signaling (*Serpina3n*, *Socs3*, *Lcn2*) were differentially expressed in the hypothalamus (Figure 6).

**Figure 7:**
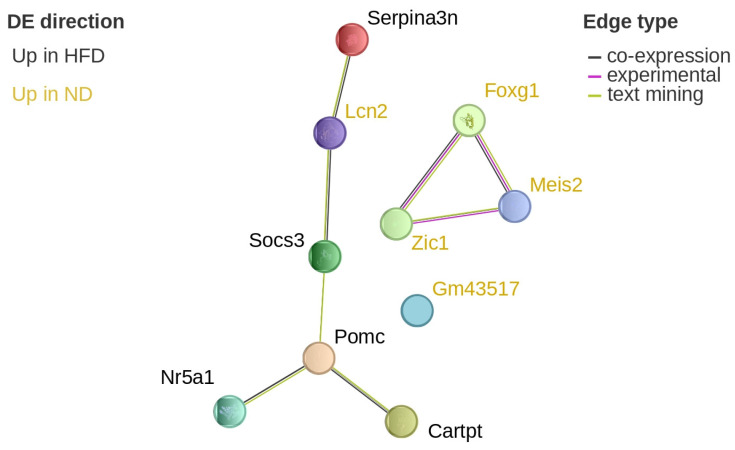
Protein network visualization of hypothalamic DEGs after 12 weeks. Visualization of the differentially expressed, potential diet-associated genes on the STRING website after 12 weeks of diet in the hypothalamus. Genes in ochre are upregulated in normal diet, while black genes are upregulated in high-fat diet. The colour of the edges denotes the source of the interaction information: black is co-expression, magenta is experimentally verified, and green is text-mining. *Serpina3n*: serine (or cysteine) peptidase inhibitor, clade A, member 3N; *Lcn2*: lipocalin-2; *Socs3*: suppressor of cytokine signaling 3; *Pomc*: pro-opiomelanocortin-alpha; *Cartpt*: CART prepropeptide; *Nr5a1*: nuclear receptor subfamily 5 group A member 1; *Gm43517*; *Zic1*: Zic family zinc finger 1; *Meis2*: Meis homeobox 2; *Foxg1*: forkhead box protein g1.

Serine (or cysteine) peptidase inhibitor, clade A, member 3N (*Serpina3n*), pro-opiomelanocortin-alpha (*Pomc*), CART prepropeptide (*Cartpt*), and suppressor of cytokine signaling 3 (*Socs3*) were upregulated in HFD-fed mice compared to ND-fed controls, with increases ranging from approximately 1.8- to 2.1-fold. Lipocalin-2 (*Lcn2*) was downregulated, showing roughly a 32-fold decrease in HFD compared to ND. STRING network analysis revealed that these DEGs were functionally connected and co-expressed (Figure 7).

**Figure 8:**
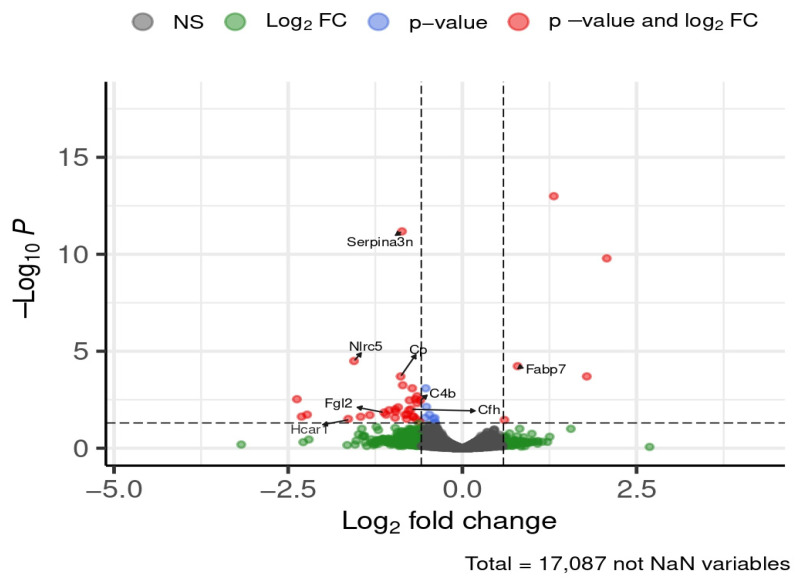
Selected DEGs of interest in the hypothalamus after 24 weeks of diet. Dashed lines mark thresholds for significance (horizontal) and fold-change (vertical).

At 24 weeks of diet exposure, eight genes of interest were differentially expressed in the hypothalamus (Figure 8).

**Figure 9:**
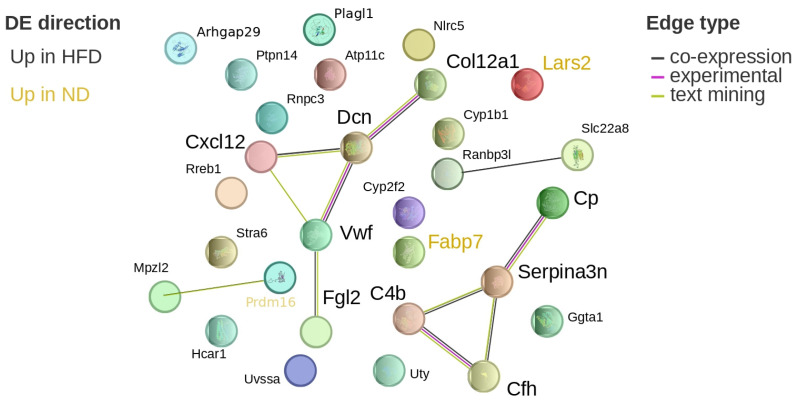
Protein network visualization of hypothalamic DEGs after 24 weeks. Visualization of the differentially expressed, potential diet-associated genes on the STRING website after 24 weeks of diet in the hypothalamus. Genes in ochre are upregulated in normal diet, while black genes are upregulated in high-fat diet. The colour of the edges denotes the source of the interaction information: black is co-expression, magenta is experimentally verified, and green is text-mining. *Cp*: ceruloplasmin; *Serpina3n*: serine (or cysteine) peptidase inhibitor, clade A, member 3N; *C4b*: complement C4B; *Cfh*: complement component factor H.

*Serpina3n*, NLR family, CARD domain containing 5 (*Nlrc5*), ceruloplasmin (*Cp*), complement C4B (*C4b*), complement component factor H (*Cfh*), fibrinogen-like protein 2 (*Fgl2*), and hydroxycarboxylic acid receptor 1 (*Hcar1*) were upregulated in HFD-fed mice, showing 1.5- to 3-fold increases. Fatty acid binding protein 7 (*Fabp7*) was downregulated by approximately 1.7-fold. STRING analysis indicated that *Cp*, *Serpina3n*, *C4b*, and *Cfh* formed a connected network (Figure 9).

#### 3.3.2. Hippocampus

**Figure 10:**
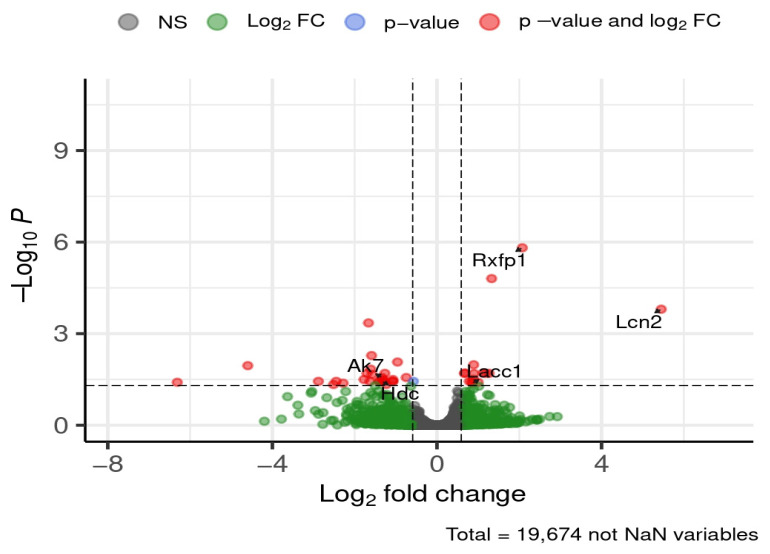
Selected DEGs of interest in the hippocampus after 12 weeks of diet. Dashed lines mark thresholds for significance (horizontal) and fold-change (vertical).

After 12 weeks of diet exposure, five genes of interest were differentially expressed in the hippocampus (Figure 10).

Relaxin/insulin-like family peptide receptor 1 (*Rxfp1*) and *Lcn2* were downregulated in HFD-fed mice compared to ND, showing ~4-fold and ~43-fold decreases, respectively. Laccase domain containing 1 (*Lacc1*) expression was also reduced in HFD-fed mice, with a ~1.8-fold decrease. In contrast, adenylate kinase 7 (*Ak7*) and histidine decarboxylase (*Hdc*) were upregulated, each increasing by ~2.5-fold.

**Figure 11:**
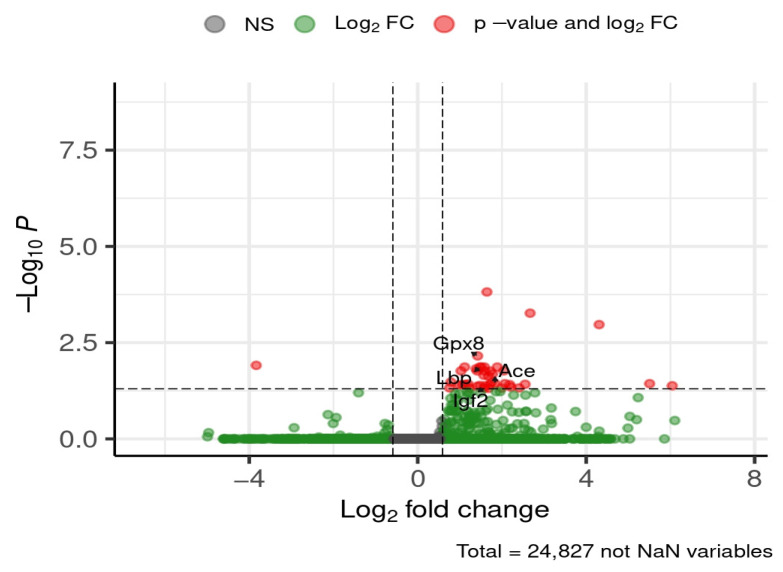
Selected DEGs of interest in the hippocampus after 24 weeks of diet. Dashed lines mark thresholds for significance (horizontal) and fold-change (vertical).

By the later time point of 24 weeks, the following selected genes were downregulated in HFD-fed mice compared to ND, with fold changes ranging from ~2.7-fold (*Gpx8*) to ~3.6-fold (*Igf2*): glutathione peroxidase 8 (*Gpx8*), lipopolysaccharide-binding protein (*Lbp*), angiotensin I converting enzyme (*Ace*), and insulin-like growth factor 2 (*Igf2*) (Figure 11).

#### 3.3.3. Cortex

**Figure 12:**
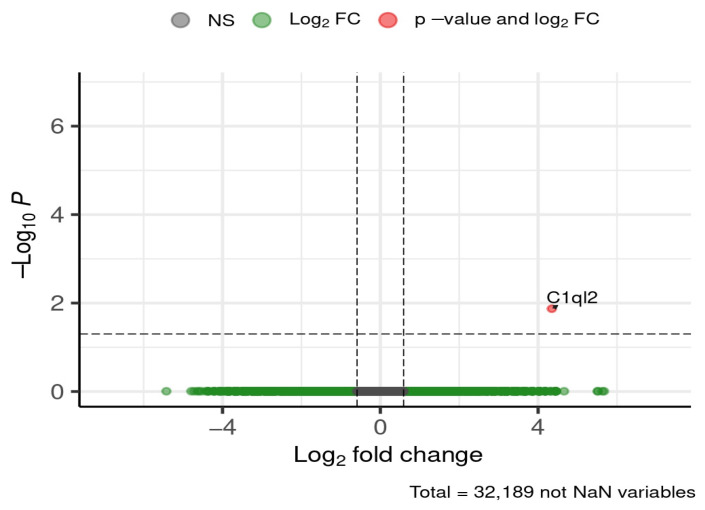
Selected DEGs of interest in the cortex after 4 weeks of diet. Dashed lines mark thresholds for significance (horizontal) and fold-change (vertical).

At the early stage of 4 weeks, a single gene of interest was differentially expressed in the cortex (Figure 12).

Complement component 1, q subcomponent-like 2 (*C1ql2*) was downregulated in HFD-fed mice compared to ND, showing a ~20-fold decrease.

**Figure 13:**
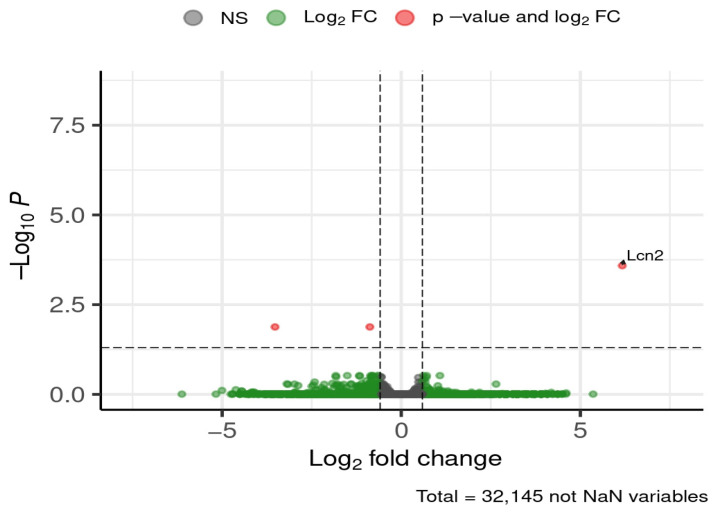
Selected DEGs of interest in the cortex after 12 weeks of diet. Dashed lines mark thresholds for significance (horizontal) and fold-change (vertical).

By 12 weeks, another gene of interest emerged as significantly altered (Figure 13).

*Lcn2* was strongly downregulated in HFD-fed mice, with a ~72-fold reduction relative to ND controls.

**Figure 14:**
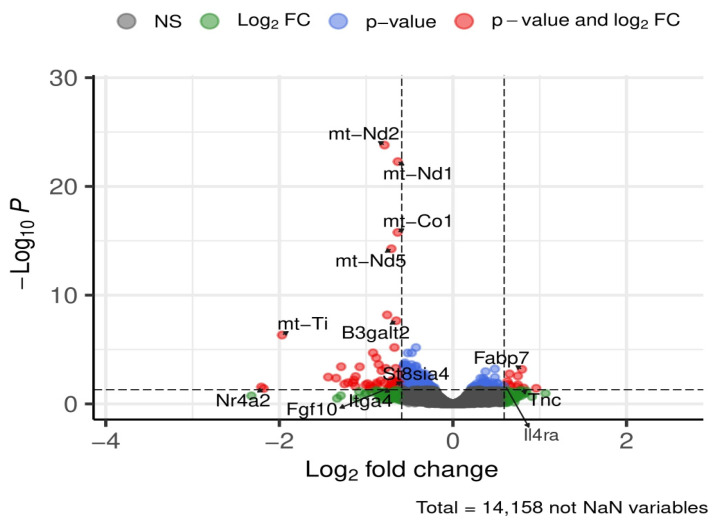
Selected DEGs of interest in the cortex after 24 weeks of diet. Dashed lines mark thresholds for significance (horizontal) and fold-change (vertical).

At 24 weeks, 13 genes of interest were affected in the cortex (Figure 14).

Genes upregulated in HFD-fed mice included beta-1,3-galactosyltransferase polypeptide 2 (*B3galt2*), ST8 alpha-N-acetyl-neuraminide alpha-2,8-sialyltransferase 4 (*St8sia4*), fibroblast growth factor 10 (*Fgf10*), nuclear receptor subfamily 4, group A, member 2 (*Nr4a2*), and integrin alpha 4 (*Itga4*), These changes reflected modest increases ranging from ~1.5-fold (*B3galt2*) to ~4.5-fold (*Nr4a2*).

Conversely, a subset of genes was downregulated under HFD, including *Fabp7*, interleukin 4 receptor, alpha (*Il4ra*), and tenascin C (*Tnc*), with decreases between ~1.5-fold and ~1.77-fold.

Additionally, several significantly regulated mitochondrial genes (*mt-Nd1*, *mt-Nd2*, *mt-Nd5*, *mt-Co1*, *mt-Ti*) were upregulated, showing increases from ~1.5-fold to ~4-fold.

#### 3.3.4. Cerebellum

No differentially expressed genes of interest were identified in the cerebellum, indicating that the transcriptomic effects of HFD were primarily localized to the hypothalamus, hippocampus, and cortex.

Together, these findings suggest that although cytokine transcripts themselves were not differentially regulated, prolonged HFD exposure induced broader transcriptional shifts consistent with microglial activation. The observed expression patterns point toward engagement of inflammatory signaling pathways—potentially including NF-κB-related processes—but likely at a lower magnitude than that typically observed in models of genetic knockdown, ischemic stroke, or LPS stimulation.

## 4. Discussion

In this study, we confirmed that male mice fed an HFD gained significantly more weight over time compared to ND-fed mice. We then asked whether this systemic dietary effect would be reflected in transcriptomic changes indicative of inflammation within distinct brain regions.

At first glance, the PCA of the specific brain regions showed that diet and exposure duration explained only a limited proportion of the overall variance. Consistent with this finding, no common signaling pathway was uniformly regulated across all brain regions, underscoring the regional specificity of diet-induced transcriptional changes. Across the entire dataset, only three genes were consistently differentially expressed between ND and HFD: *Lcn2*, *Ch25h*, and *Gimap9*.

LCN2 has been described as an adipokine involved in glucose and lipid homeostasis, and as an acute phase mediator in the CNS [41,42]. Prior studies have reported opposing effects of LCN2. For example, LCN2-deficient mice are cold-sensitive and develop significantly increased body fat mass as well as exacerbated dyslipidemia, fatty liver, and insulin resistance when fed HFD compared to wild-type (WT) mice [42]. LCN2 can exert anti-inflammatory effects after LPS stimulation, possibly by controlling the activation of the NF-κB-STAT3 loop and thus limiting the inflammatory response [41,43]. In contrast, other studies describe LCN2 as pro-inflammatory, showing that its expression is induced after injury and promotes hippocampal microglial activation and conversion to M1 phenotype [44,45]. LCN2 plays an important role in HFD-induced adipose tissue remodeling as well as in inflammation, especially in the brain. In our dataset, *Lcn2* was strongly downregulated under HFD compared to ND, suggesting that its potential regulatory, anti-inflammatory functions [41,43] may have been diminished in this context. By contrast, the pronounced pro-inflammatory effects observed in stroke or acute injury [44,45] were not reproduced by dietary stress alone.

CH25H catalyzes the oxidation of cholesterol into 25-hydroxycholesterol (25-HC), a soluble metabolite involved in lipid metabolism and inflammatory signaling. Previous studies have reported pro-inflammatory functions of CH25H, including elevated expression in adipose tissue in response to HFD [46]. Contrarily, a distinct CH25H+ microglia cluster has been identified in the ischemic brain, where it may exert protective functions [47]. The downregulation of *Ch25h* in our study may therefore reflect a reduced capacity for microglial protective responses under HFD, potentially tilting the balance toward vulnerability in neurodegenerative settings.

While our study provides insight into the effects of HFD on microglial activation and transcriptional changes, several limitations should be noted. First, the sample size (*n* = 3 per group per time point) is relatively modest for RNA-seq studies involving multiple brain regions. Although *DESeq2* is robust for small sample sizes, this limited group size may reduce the ability to detect subtle transcriptional differences and fully capture inter-individual biological variability. Importantly, however, the three biological replicates per group showed highly consistent expression profiles with no apparent outliers (see correlation matrix, Supplementary Figure S3), supporting the reliability of the observed transcriptional patterns despite the small sample size.

Second, the HFD used in this study was composed primarily of medium-chain saturated fatty acids (C12:0 14.96%, C14:0 5.75%, C16:0 3.14%), with smaller amounts of monounsaturated (C18:1 2.86%) and polyunsaturated (C18:2 1.82%, C18:3 0.15%) fats (Supplementary Data S4). The predominance of saturated fats may contribute to the observed microglial activation and selective transcriptional changes, consistent with previous studies linking dietary saturated fats to neuroinflammatory signaling.

Third, while our findings support a link between HFD exposure, microglial activation, and NF-κB signaling, additional experimental approaches—such as pathway inhibition, genetic manipulations, qPCR, or immunohistochemistry—would provide more definitive mechanistic evidence. These additional approaches were beyond the scope of the present study, which aimed to characterize transcriptional and cellular responses to HFD in vivo.

Fourth, the conclusions rely exclusively on transcriptomic data, and the link between gene expression changes and microglial activation remains inferential. Nevertheless, protein–protein interaction analysis using STRING identified connected networks among several DEGs of interest (notably complement and acute-phase components), indicating potential functional interactions. These observations provide a basis for future pathway-level enrichment analyses and targeted mechanistic validation.

Finally, RNA-seq was performed on bulk tissue rather than isolated microglia, so transcriptional contributions from neurons, astrocytes, or other cell types cannot be excluded. Importantly, the key DEGs identified here are known to be involved in microglial activation. This supports the interpretation that the observed transcriptional changes primarily reflect microglial responses, albeit reflecting decreased activity or regulatory function rather than upregulation. Future studies using microglia-specific isolation or single-cell RNA-seq approaches will be valuable to further validate these findings.

Collectively, these global observations suggest that HFD does not drive broad transcriptional remodeling across all brain regions but rather induces selective changes in a limited number of inflammation- and lipid-related genes. The following sections examine these alterations in more detail within the hypothalamus, hippocampus, and cortex, highlighting region-specific signatures of microglial activation and inflammatory signaling.

### 4.1. Hypothalamus

In the hypothalamus, *Serpina3n* was upregulated, consistent with its established role in diet-induced hypothalamic inflammation [48]. Diet-induced obesity is also associated with dysfunction of POMC neurons, a hypothalamic cell population critical for maintaining energy balance by regulating food intake and energy expenditure [49]. *Socs3*, which was elevated in our study, is known to increase in chronic obesity and is widely considered a mediator of obesity-associated leptin resistance [50]. Similarly, *Cartpt* was upregulated, and previous publications have shown that its expression is highly sensitive to metabolic changes [51,52,53].

At 24 weeks of HFD exposure, several additional genes related to inflammatory signaling were differentially expressed. *Nlrc5* was upregulated, promoting microglia-mediated inflammation through NF-κB and AKT pathways [54]. The acute-phase protein *Cp* was also increased, which is induced during inflammation and has been detected at elevated levels in the brains of patients with neurodegenerative diseases [55]. Components of the complement cascade were altered as well: *C4b* was upregulated, supporting prior evidence that complement activation contributes to AD pathogenesis [56], while *Cfh*, a key negative regulator of the complement cascade, was also induced [57].

Other immune-related transcripts showed parallel changes. *Fgl2*, an important pleiotropic immunomodulatory cytokine, was upregulated, consistent with its activation of downstream inflammatory pathways in inflammatory diseases and cancer [58,59]. *Hcar1*, first described in adipose tissue as an inhibitor of lipolysis but in the brain known to stimulate neurogenesis and microglial activation, was likewise upregulated [60].

In contrast, *Fabp7* was downregulated in HFD compared to ND. This astrocyte-enriched gene has dichotomous effects in regulating both pro- and anti-inflammatory signaling pathways via the regulation of cyclooxygenase-2 (COX-2) [61].

Overall, these findings indicate that the hypothalamus mounts a robust transcriptional response to long-term HFD exposure. The gene expression profile suggests that hypothalamic microglia may act as early responders, with inflammatory processes potentially initiated through complement activation and transcriptional changes consistent with involvement of NF-κB-related signaling. However, these interpretations are based on transcriptomic patterns rather than direct measurements of pathway activation.

### 4.2. Hippocampus

In contrast to the hypothalamus, where early inflammatory signaling changes were observed, the hippocampus displayed a distinct set of transcriptional alterations under HFD. Alongside the downregulation of *Lcn2*, we observed reduced expression of *Rxfp1* and *Lacc1*. RXFP1 has been reported to attenuate hippocampal inflammation by inhibiting NF-κB activity via the PI3K-AKT/TNFAIP3 signaling pathway [62]. Likewise, LACC1 functions in an anti-inflammatory capacity, promoting the production of TNF-α and interleukin-17 (IL-17) [63], and potentially suppressing the production of pro-inflammatory cytokines through the downstream polyamine pathway [64].

On the other hand, *Ak7* and *Hdc* were upregulated under HFD compared to ND. AK7 may promote the overproduction of pro-inflammatory cytokines by microglia through elevated NF-κB activity, which in turn increases IL-1β and TNF-α expression [65]. HDC plays a complex role: Knockout studies show that while microglia numbers remain unchanged, the density of their ramifications is reduced [66], and furthermore, the production of anti-inflammatory factors is diminished [67].

After 24 weeks of HFD, several additional genes with protective or homeostatic functions were significantly downregulated. GPX8 is an enzyme, supporting cellular defense against oxidative stress [68]. LBP interacts with TLR4 to regulate TNF-α production and contributes to neuroprotection via the p38 mitogen-activated protein kinase (MAPK) signaling pathway [69]. IGF2 inhibits microglial over-activation and prevents their conversion to a pro-inflammatory phenotype, thereby protecting hippocampal neurogenesis [70]. ACE enhances microglial immune functions, and ACE-expressing microglia have potential utility as a cell-based therapy to improve endogenous microglial responses to nAβ in AD [71].

In summary, these findings indicate that in the hippocampus, genes associated with microglial protection and anti-inflammatory activity were consistently downregulated, while genes with pro-inflammatory functions were upregulated. Similarly to the hypothalamus, several of these transcriptional shifts are compatible with engagement of NF-κB-related signaling pathways.

### 4.3. Cortex

In the cortex, transcriptional changes emerged at multiple time points under HFD. After 4 weeks, *C1ql2* was downregulated more than 20-fold. C1QL2, a component of the complement system, interacts with brain-specific angiogenesis inhibitor 3, BAI3/ADGRB3, and contributes to synapse formation and maintenance, suggesting early synaptic effects of HFD exposure [72].

After 12 weeks, *Lcn2* expression was reduced by over 72-fold, consistent with its downregulation in other brain regions.

By 24 weeks, 13 genes of interest exhibited significant differential expression. Notably, several mitochondrial genes were upregulated in HFD-fed mice. The mitochondrially encoded NADH-ubiquinone oxidoreductase core subunits 1, 2, and 5 (mt-Nd1, mt-Nd2, mt-Nd5) are subunits of mitochondrial respiratory complex I, whereas the mitochondrially encoded cytochrome c oxidase 1 (mt-Co1) is a subunit of the enzyme cytochrome c oxidase or complex IV. Mitochondrial dysfunction in the brain is associated with neuroinflammation and oxidative stress [73] and occurs early in all major neurodegenerative diseases such as AD [74]. Furthermore, there is strong evidence that this dysfunction plays a causal role in disease pathogenesis [74]. Upregulation of mitochondrial respiratory chain genes under HFD may reflect a compensatory attempt to sustain energy production despite metabolic stress [2,75]. Given that complex I is a major source of reactive oxygen species [76], these changes may contribute to oxidative imbalance. The upregulation of the mitochondrially encoded tRNA isoleucine (mt-Ti) gene is less clear. However, its expression can be affected by the overall changes in mitochondrial gene expression and function caused by HFD.

Among the remaining upregulated genes, several are linked to inflammatory or microglia-related pathways. *B3galt2* has been associated with inflammatory signaling, while knockdown of B3GALT2 reduces TNF-α and IL-6 levels via the TLR4/NF-κB pathway [77]. *St8sia4* encodes an enzyme responsible for polysialylation of E-selectin ligand-1 (ESL-1) and neuropilin 2 (NRP2) in microglia [78]. The polySia-NRP2 and polySia-ESL-1, located in the Golgi compartment, emerged during injury-induced activation of mouse microglia, and inflammatory activation by stimulation with LPS led to their translocation to the cell surface [79,80].

It has been observed that *Fgf10* expression is increased after spinal cord injury (SCI). Activated microglia produce a variety of pro-inflammatory mediators after SCI. FGF10 treatment decreases microglial activation significantly in vivo as well as TLR4-expression, leading to reduced p-IκB-α and IκB-α degradation and nuclear translocation of NF-κB transcription factors. In summary, this suggests that the TLR4/NF-κB signaling pathway is involved in the underlying anti-inflammatory mechanism of FGF10 [81]. *Nr4a2* is associated with Parkinson’s disease (PD) and AD [82] as well as in synovium and cartilage from patients with RA, psoriatic arthritis, and osteoarthritis [83]. Co-expression between NR4A2 and Aß has been found in glutamatergic neurons of the frontal cortex, among others [82]. Furthermore, it is upregulated and active in arthritis during disease progression, and it may be a promising target downstream of TNF-α and NF-κB signaling pathways [83]. *Itga4* is expressed in border-associated macrophages, which possibly allows them to migrate and adhere to their specific locations [84].

In contrast, several genes were downregulated in the cortex after 24 weeks of HFD. These included *Fabp7*, which was already downregulated in the hypothalamus and hippocampus and described above, respectively. Interleukin-4 (IL-4) influences neurogenesis through microglia and inflammation through direct signaling in neurons. The downregulation of *Il4ra* results in reduced IL4 receptor signaling in microglia [85]. *Tnc* regulates chemotaxis, phagocytosis, and pro-inflammatory cytokine production in microglia as an endogenous activator of TLR4 [86]. At least, TNC induces histone-deacetylase 1 (HDAC1) expression in microglia [87].

### 4.4. Cerebellum

Examination of the cerebellum revealed no DEGs beyond the early time point associated with microglial activation or inflammatory signaling under HFD. This absence of significant transcriptional changes suggests that, unlike the hypothalamus, hippocampus, and cortex, the cerebellum is relatively resistant to diet-induced inflammatory signaling at the mRNA level. All identified DEGs, which did not meet criteria for microglial or inflammatory relevance, are provided in the Supplementary Data S1–S3.

## 5. Conclusions

Taken together, our results indicate that genes associated with microglial activation and inflammatory signaling were differentially expressed in multiple brain regions, particularly after 24 weeks of HFD. These findings demonstrate that prolonged high-fat consumption can impact brain homeostasis, although some inconsistencies remain. Notably, several globally regulated genes, such as *Lcn2* and *Ch25h*, exhibit context-dependent pro- and anti-inflammatory effects, reflecting the complexity of diet-induced transcriptional regulation.

A key observation emerging from our analysis is that several of the DEGs align with components or downstream targets of the NF-κB signaling pathway, suggesting that HFD may influence NF-κB-related inflammatory processes across regions. Previous studies have linked HFD-related obesity to inflammatory microglia and COX-2 activation. When analyzing the identified DEGs, we observed regulation via the NF-κB signaling pathway on several occasions. It has been shown that activation of the NF-κB pathway stimulates COX-2 production, leading to inflammation [88], and even that COX-2 gene expression is mainly regulated by NF-κB [89,90]. Moreover, phytosterols (PS) have been reported to inhibit COX-2, thereby exerting anti-inflammatory effects [91]. We propose that supplementing obesity-associated HFD with physiological levels of PS could modulate the activation of the arachidonic acid cascade and thus the formation of pro-inflammatory bioactive lipids and their metabolites in the brain, with beneficial effects on microglial cells and overall neuroinflammatory status. This hypothesis needs to be investigated in further experiments.

## Figures and Tables

**Table 1:** Group allocation of male mice for RNA-sequencing data analysis.

	Male			
	4 Weeks	12 Weeks	24 Weeks	Sum ∑
**ND**	3	3	3	9
**HFD**	3	3	3	9
**Sum ∑**	6	6	6	**18**

## Data Availability

The mapped RNA-seq data on which this manuscript is based are available in the European Nucleotide Archive (ENA) (https://www.ebi.ac.uk/ena/browser/home) under the project accession PRJEB104708. All additional processed data are included in the article/Supplementary Material. Further inquiries can be directed to the corresponding authors.

## Supplementary Materials

The following supporting information can be downloaded at: https://www.mdpi.com/article/10.3390/brainsci16010029/s1, Supplementary Data S1: All identified DEGs separated by examined brain regions after 4 weeks; Supplementary Data S2: All identified DEGs separated by examined brain regions after 12 weeks; Supplementary Data S3: All identified DEGs separated by examined brain regions after 24 weeks; Supplementary Data S4: Diet composition; Supplementary Figure S1: Heatmap with all samples; Supplementary Figure S2: Heatmap with all samples, genes of interest discussed in the manuscript; Supplementary Figure S3: Correlation matrix of all samples.

## Abbreviations

The following abbreviations are used in this manuscript:

25-HC	25-hydroxycholesterol
Ace	Angiotensin I converting enzyme
AD	Alzheimer’s disease
Ak7	Adenylate kinase 7
AKT	Protein kinase B
AML	Acute myeloid leukemia
API	Application Programming Interface
B3galt2	Beta-1,3-galactosyltransferase polypeptide 2
C1ql2	Complement component 1, q subcomponent-like 2
C4b	Complement C4B
Cartpt	CART prepropeptide
cDNA	Complementary DNA
Cfh	Complement component factor H
Ch25h	Cholesterol 25-hydroxylase
CNS	Central nervous system
COX-2	Cyclooxygenase-2
Cp	Ceruloplasmin
DE	Differential expression
DEGs	Differentially expressed genes
DFG	Deutsche Forschungsgemeinschaft
ENA	European Nucleotide Archive
ESL-1	E-selectin ligand-1
Fabp7	Fatty acid binding protein 7
Fgf10	Fibroblast growth factor 10
Fgl2	Fibrinogen-like protein 2
Gimap9	IMAP family member 9
Gpx8	Glutathione peroxidase 8
Hcar1	Hydroxycarboxylic acid receptor 1
HDAC1	Histone-deacetylase 1
Hdc	Histidine decarboxylase
HFD	High-fat diet
Igf2	Insulin-like growth factor 2
IL-1β	Interleukin-1 beta
IL-4	Interleukin-4
IL-6	Interleukin-6
IL-17	Interleukin-17
Il4ra	Interleukin 4 receptor, alpha
Itga4	Integrin alpha 4
Lacc1	Laccase domain containing 1
Lbp	Lipopolysaccharide-binding protein
Lcn2	Lipocalin-2
MAPK	Mitogen-activated protein kinase
MMP-12	Matrix metallopeptidase 12
MS	Multiple sclerosis
Mt-Co1	Mitochondrially encoded cytochrome c oxidase 1
Mt-Nd1	Mitochondrially encoded NADH-ubiquinone oxidoreductase core subunit 1
Mt-Nd2	Mitochondrially encoded NADH-ubiquinone oxidoreductase core subunit 2
Mt-Nd5	Mitochondrially encoded NADH-ubiquinone oxidoreductase core subunit 5
Mt-Ti	Mitochondrially encoded tRNA isoleucine
NADH	Nicotinamide adenine dinucleotide hydride
NCS	NextSeq 2000 Control Software
ND	Normal diet
NF-κB	Nuclear factor kappa-B
Nlrc5	NLR family, CARD domain containing 5
NO	Nitric oxide
Nr4a2	Nuclear receptor subfamily 4, group A, member 2
NRP2	Neuropilin 2
PCA	Principal component analysis
PD	Parkinson’s disease
PI3K	phosphatidylinositol 3′–kinase
Pomc	Proopiomelanocortin-alpha
PS	Phytosterols
RNA	Ribonucleic acid
ROS	Reactive oxygen species
RTA	Real Time Analysis Software
Rxfp1	Relaxin/insulin-like family peptide receptor 1
SCI	Spinal cord injury
Serpina3n	Serine (or cysteine) peptidase inhibitor, clade A, member 3N
Socs3	Suppressor of cytokine signaling 3
SR	Single-read
St8sia4	ST8 alpha-N-acetyl-neuraminide alpha-2,8-sialyltransferase 4
STAT3	Signal transducer and activator of transcription 3
TGFβ1	Transforming growth factor beta-1
TLR4	Toll-like receptor 4
Tnc	Tenascin C
TNF-α	Tumor necrosis factor alpha
tRNA	Transfer RNA
WT	Wild-type
