# Supplementary material for: Diet-Driven Microglial Activation: Region-Specific Neuroinflammation in the Mouse Brain
Source: Brain Sci. 2025 Dec 25;16(1):29. doi: 10.3390/brainsci16010029 (PMC12839311; doi:10.3390/brainsci16010029)
Supplement: Supplementary file 1 [file brainsci-16-00029-s001.zip › Supplementary Files/Supplementary Data S4_Diet composition.pdf]

ssniff® EF R/M D12331 mod.* / Surwit

Experimental diet with very high fat content (hydrogenated coconut oil) / DIO

Description

The experimental diet is characterized by extremely high amounts of fat with middle-chain, saturated fatty acids (coconut oil); because of its high fat content the feed will quickly induce obesity and and may promote the development of Metabolic Syndrome with diabetes type 2 (NIDDM). The feeding period until first clinical symptoms might be observed depends largely on the rat or mouse strain and the previous dietary history (fat supply).

Crude Nutrients	[%]
Dry matter	97.1
Crude protein (N x 6.25)	21.6
**Crude fat**	**35.7**
Crude fibre	0.1
Crude ash	5.2
N free extracts	34.1
Starch	0.9
**Sugar**	**17.1**
Dextrines	16.5

[1)] *ME calculated according to the pig formula, Annex 4 of the German feed regulation*

[2)] *ME calculated with the Atwater factors*

Energy	[MJ/kg]
**Gross Energy (GE)**	**25.1**
**Metabolizable Energy (ME)**	

22.6 [1)]

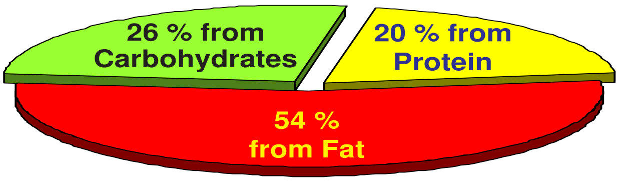

22.8 [2)]

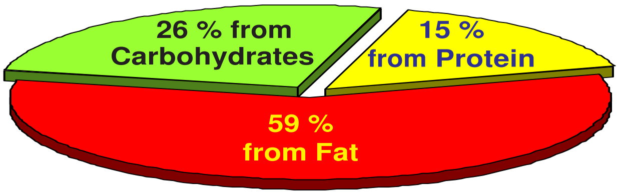

Minerals	[%]
Calcium	0.86
Phosphorus	0.60
Sodium	0.34
Magnesium	0.20
Potassium	0.90

Fatty acids	[%]
C 6:0	0.20
C 8:0	2.53
C 10:0	2.00
C 12:0	14.96
C 14:0	5.75
C 16:0	3.14
C 16:1	0.02
C 17:0	—
C 18:0	1.05
C 18:1	2.86
C 18:2	1.82
C 18:3	0.15
C 20:0	0.01
C 20:1	0.01
C 20:5	—
C 22:6	—
Cholesterol [mg/kg]	—

Amino acids	[%]
Lysine	1.64
Methionine	0.81
Cystine	0.19
Met+Cys	1.00
Threonine	0.89
Tryptophan	0.26
Arginine	0.73
Histidine	0.63
Valine	1.36
Isoleucine	1.04
Leucine	1.96
Phenylalanine	1.07
Phe+Tyr	2.13
Glycine	0.42
Glutamic acid	4.49
Aspartic acid	1.48
Proline	2.29
Alanine	0.65
Serine	1.19

Vitamins	per kg
Vitamin A	15,000 IU
Vitamin D_3	1,500 IU
Vitamin E	154 mg
Vitamin K (as menadione)	20 mg
Vitamin C	30 mg
Thiamin (B_1)	16 mg
Riboflavin (B_2)	16 mg
Pyridoxine (B_6)	17 mg
Cobalamin (B_{12})	30 µg
Nicotinic acid	46 mg
Pantothenic acid	55 mg
Folic acid	19 mg
Biotin	310 µg
Choline-Chloride	1,050 mg
Inositol	80 mg

Trace elements	per kg
Iron	151 mg
Manganese	90 mg
Zinc	60 mg
Copper	13 mg
Iodine	1.05 mg
Selenium	0.14 mg
Cobalt	0.14 mg

Feed composition
On request

Main products

E15772-30 Meal
E15772-34 10 mm pellets
(orange colour)

Production and sale
ssniff Spezialdiäten GmbH
Phone:+49-(0)2921-9658-0
Fax: +49-(0)2921-9658-40
E-Mail mail@ssniff.de
www.ssniff.de / www.ssniff.com

* *Research Diets Inc.*
