# Supplementary figures and images for: Diet-Driven Microglial Activation: Region-Specific Neuroinflammation in the Mouse Brain
Source: Brain Sci. 2025 Dec 25;16(1):29. doi: 10.3390/brainsci16010029 (PMC12839311; doi:10.3390/brainsci16010029)

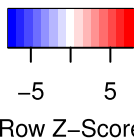

**Location**

- cerebellum
- cortex
- hippocampus
- hypothalamus

**Diet**

- HFD
- ND

**Duration**

- 4 weeks
- 12 weeks
- 24 weeks

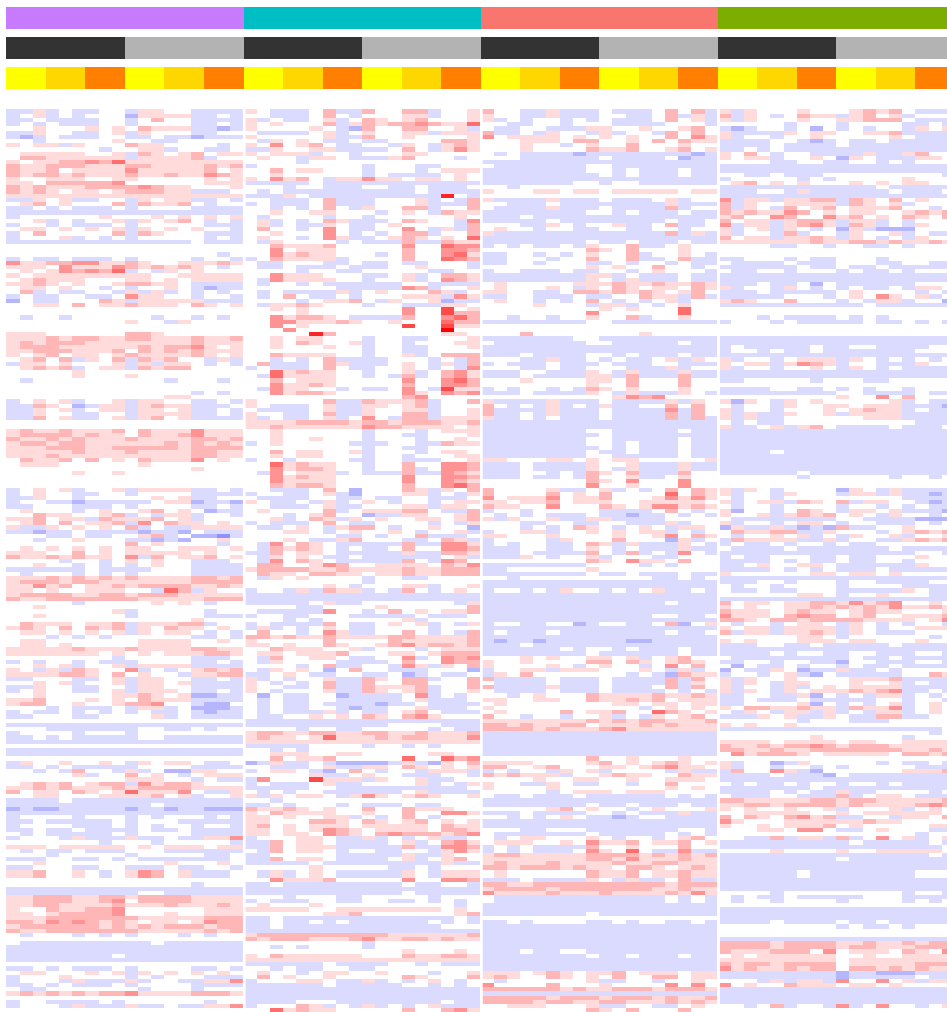

Supplement: Supplementary file 1 [file brainsci-16-00029-s001.zip › Supplementary Files/Supplementary Figure S1.pdf]

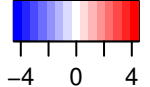

### Location

- cerebellum
- cortex
- hippocampus
- hypothalamus

### Diet

- HFD
- ND

### Duration

- 4 weeks
- 12 weeks
- 24 weeks

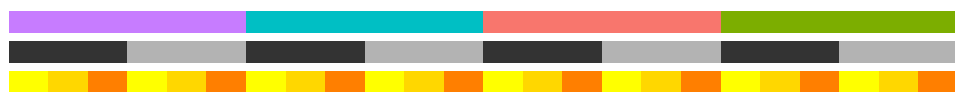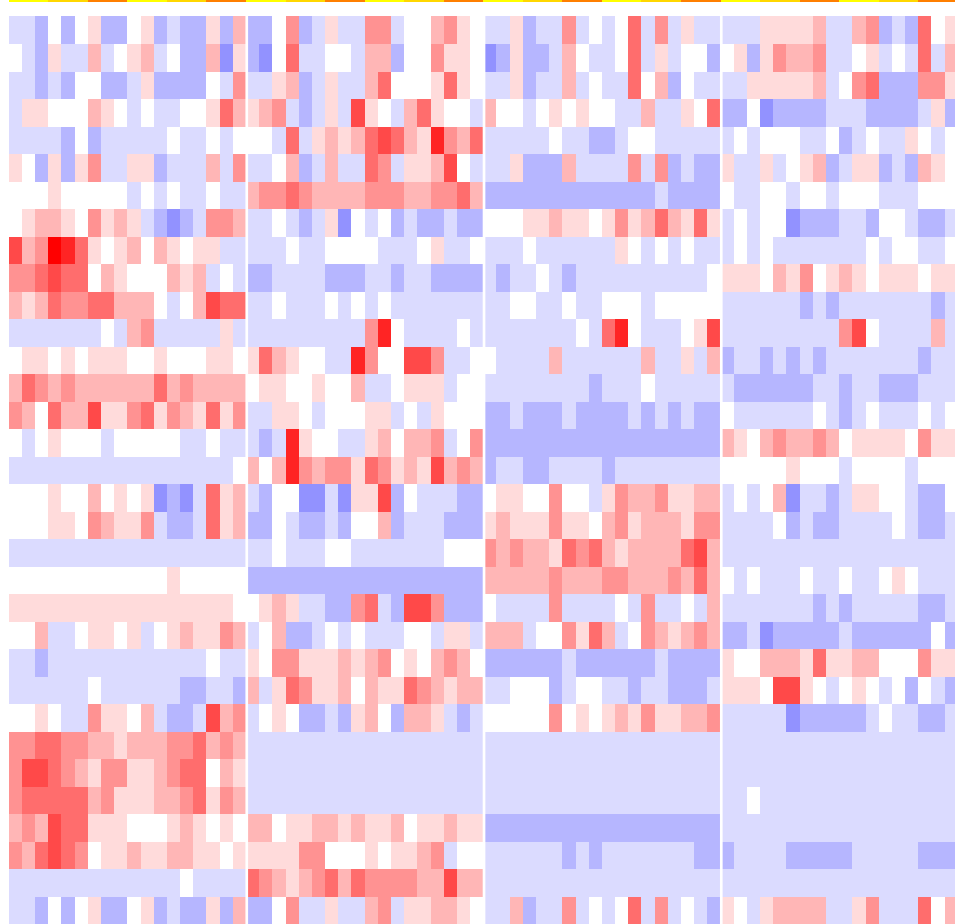

Supplement: Supplementary file 1 [file brainsci-16-00029-s001.zip › Supplementary Files/Supplementary Figure S2.pdf]

# Correlation of RNAseq experiments

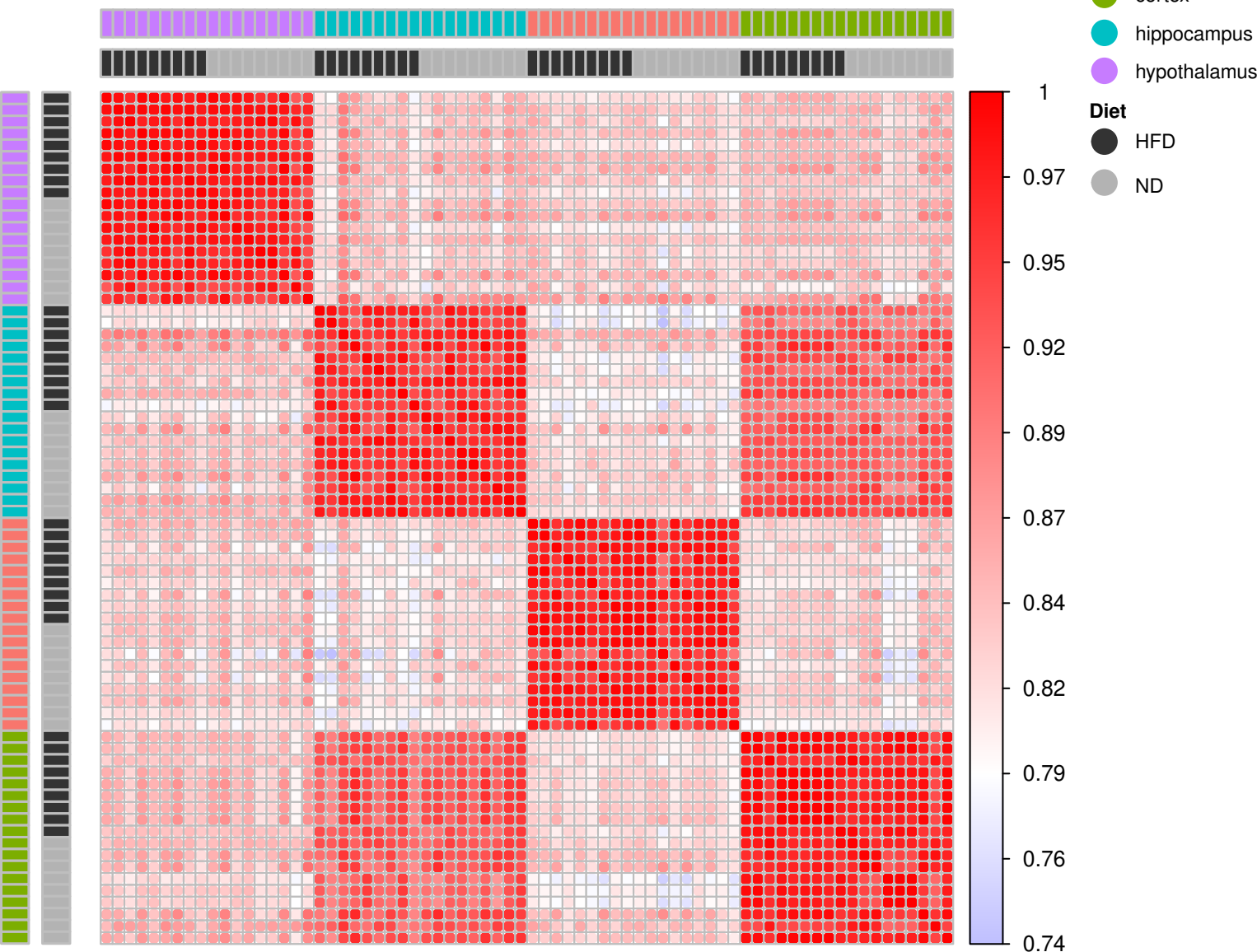

Supplement: Supplementary file 1 [file brainsci-16-00029-s001.zip › Supplementary Files/Supplementary Figure S3.pdf]
